# Effect of pH and Particle Charge on the Interfacial Properties of Biocatalytic Pickering Emulsions—Where Are the Enzymes Located?

**DOI:** 10.1021/acs.langmuir.5c02459

**Published:** 2025-09-08

**Authors:** Maximilian Seiler, Maria Loidolt-Krüger, Regine von Klitzing, Anja Drews

**Affiliations:** † Department of Chemical and Process Engineering, Technical University Berlin, Ackerstraße 76, 13355 Berlin, Germany; ‡ Process Engineering in Life Science Engineering, HTW Berlin, Wilhelminenhofstraße 75 A, 12459 Berlin, Germany; § Picoquant, Rudower Chaussee 29 (IGZ), 12489 Berlin, Germany; ∥ Department of Physics, Soft Matter at Interfaces, Technical University Darmstadt, 64289 Darmstadt, Germany

## Abstract

Pickering emulsions (PEs), where water-in-oil (w/o) droplets are stabilized by nanoparticles (NPs), offer a promising platform for biocatalysis by providing a large interfacial area crucial for efficient substrate conversion. While several lipase catalyzed reactions in PEs have been demonstrated, the exact interfacial structure is unknown. This study focuses on the interfacial network formed by NPs and *Candida rugosa* lipase (CRL) at the octanol/water-interface by varying pH and NP charge. By applying different methods, the location of lipases within a PE was identified and the enzyme concentration profile quantified for the first time. Positively charged nanoparticles (NP+) adsorbed at the o/w-interface together with CRL to form a network-structure. The relation between individual and simultaneous adsorption showed a constant value of 0.75 for the investigated pH range. Negatively charged particles (NP-) did not adsorb spontaneously at the negatively charged octanol/water-interface and therefore showed no influence on the enzyme adsorption behavior. Interfacial shear rheology measurements further revealed distinct elastic behavior of the enzyme–particle network due to attractive interactions between positively charged nanoparticles and CRL. This was shown by a 4.4-fold increase in the interfacial storage modulus. In contrast, repulsive interactions—either between CRL and positively charged particles at low pH or with negatively charged particles—did not enhance the elastic response of CRL at the interface. Confocal laser scanning microscopy of prepared PE droplets showed an interfacial CRL layer thickness of 0.75 μm for NP+ and 0.51 μm for NP–. Using NP+ results in a 30% higher interfacial enzyme concentration, indicating a more compact layer structure. These insights contribute to optimizing biocatalytic systems using PEs for industrial applications and provide a basis for the quantitative analysis of the interfacial layer in a Pickering emulsion.

## Graphical abstract

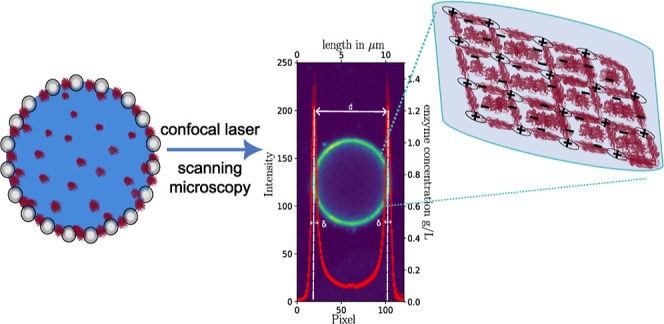

## Introduction

Lipases are among the most widely used enzymes in the chemical industry due to their high stability and substrate variety,^1^ but are mostly active when adsorbed at a hydrophobic surface, due to their interfacial activation.^2^ For biocatalytic applications, where the substrates are mostly insoluble in water, a large interfacial area between the oil and the water phase becomes a requirement for a high reaction yield. A promising way of achieving this sufficient interfacial area is to stabilize droplets of water inside oil by nanoparticle (NP) adsorption, which is called a Pickering emulsion (PE).^3^ PEs have been studied as a tool for biocatalysis for more than a decade, e.g. for esterification,^4,5^ transesterification,^6–8^ epoxidation,^9,10^ hydrolysis^11,12^ and carboligation.^13^ They are also of great interest for drug delivery systems^14^ and in the food industry.^15^

Using Pickering emulsions for biocatalysis, a distinction between Pickering assisted catalysis (PAC) and Pickering interfacial catalysis (PIC) is made.^16^ The former method utilizes particles solely for the stabilization mechanism and the enzymes encapsulated inside the droplets are either confined to the droplets’ bulk phase or adsorbed at the o/w interface. Usually, the enzymes are dissolved in the aqueous phase while the particles are dispersed in the oil phase prior to emulsification. In contrast, PIC immobilizes enzymes onto the particles by adsorption, cross-linking or covalent bonding prior to emulsification. This method holds the advantage of the catalyst being located exactly at the oil/solid interface (see Figure 1). Although having a generally higher catalytic activity than the native enzyme,^17^ an additional immobilization step is necessary, which can compromise catalytic performance.^18^

**Figure 1:**
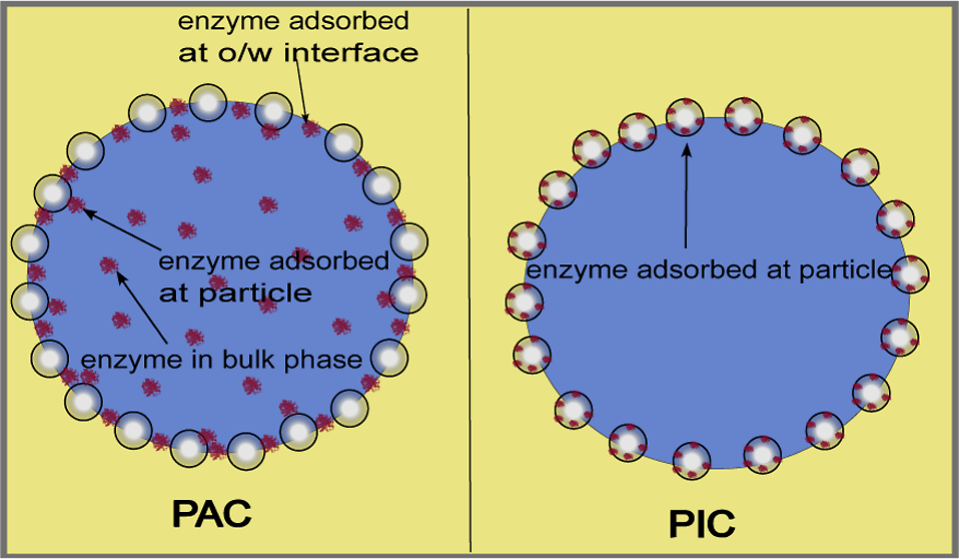
Scheme of a droplet prepared as Pickering assisted catalysis (PAC)­(left) and as Pickering interfacial catalysis (PIC)­(right).

A clear distinction between PIC and PAC becomes more challenging when the homogeneous catalyst in PAC also spontaneously adsorbs onto the particles or the liquid–liquid interface. *Candida rugosa* lipase (CRL) was chosen as it is one of the most extensively studied enzymes for biocatalytic applications^19^ and readily available in large quantities at low cost. Using lipases, the advantage of PAC is the combination of immobilization, activation and emulsification in a single step, but with the drawback of not knowing the exact location of the enzymes after PE preparation.

For efficient biocatalysis, enzymes should ideally be confined to a monolayer at the interface. The formation of thick multilayers can lead to reduced specific reaction rates due to mass transfer limitations of substrates and products, and not all enzymes may participate in the catalytic process. Moreover, understanding the spatial distribution of enzymes at the interface is essential for accurately modeling enzyme kinetics in Pickering emulsions. Therefore, controlling enzyme distribution in PAC systems is crucial.

Since many studies on Pickering assisted catalysis have not specifically focused on enzyme localization, the system is often treated as a “black box”.^5,6,20–26^ Confocal laser scanning microscopy was used in a few studies to qualitatively analyze enzyme distribution in PAC systems, typically revealing enzymes localized at the interface.^11,12,27–30^ In contrast, for PIC systems, enzymes are immobilized on particles prior to emulsification and thus primarily reside on the particles.^10,31–33^

Liu et al. calculated the adsorption of CRL at the isooctane/water interface by measuring the residual fluorescence in the aqueous phase after PE preparation. They found that only 1.3% of the signal remained, indicating that 98.7% of the enzyme adsorbed at the interface.^8^ Similarly, Wei et al. estimated the adsorbed amount of Candida antarctica lipase B on particles by measuring the residual UV–vis signal and concluded that 53.3% of the enzyme had adsorbed onto the particle surfaces.^11^

In biocatalytic applications, Pickering emulsions are predominantly water-in-oil (w/o) to retain and facilitate recyclability of enzymes. The type of emulsion—w/o or oil-in-water (o/w)—is determined by factors such as the volume fraction, particle wettability (contact angle), and the initial dispersion location of the particles.^34,35^ Therefore, the nanoparticles used in this study were made partially hydrophobic to enable dispersion in the oil phase. The particles carried either a positive or a negative charge to investigate electrostatic interactions with the lipases and have been used in previous studies.^36,37^

On one hand, the role of strong electrostatic interactions between nanoparticles and the formation of self-assembled monolayers at oil–water interfaces has been extensively investigated.^38–45^ The adsorption of charged nanoparticles at charged liquid interfaces was studied for different particle sizes, surface charge densities, contact angles, and electrolyte concentrations.^46^ For example, the addition of electrolytes can screen electrostatic interactions, thereby enhancing particle adsorption. This effect was even more pronounced at charged oil–water interfaces.^46^

While it is well established that nanoparticles adsorb strongly at fluid interfaces to minimize the system’s free energy, their effect on interfacial tension (IFT) remains a subject of ongoing debate.^47^ Some studies reported a decrease in IFT upon nanoparticle adsorption,^48–51^ whereas others found no significant change.^52–55^ More comprehensive discussions can be found in the reviews by Gholinezhad et al.^56^ and Sofla et al.,^50^ and this topic will be further addressed in the Results and Discussion section.

On the other hand, the adsorption behavior of lipases has been widely studied at liquid interfaces,^57–61^ for immobilization on solid particles,^62–68^ and within the context of Pickering interfacial catalysis.^10,31–33^ However, lipase adsorption at heterogeneous interfaces, such as particle-laden oil–water interfaces, remains largely unexplored. Variations in particle and enzyme charge induce electrostatic repulsion or attraction, thereby potentially influencing enzyme activity, droplet size, mass transfer, and PE stability.

Previous experiments showed the influence of pH, buffer strength and particle charge on the activity of *C. rugosa* lipase (CRL) as well as on the drop sizes of the PE.^36^ Furthermore, the energy input during the preparation of Pickering emulsions significantly influences the resulting droplet size^69^ and the composition of the interface. Since characterizing the interfacial structure requires well-defined and undisturbed interfaces, introducing additional energy during observation poses considerable challenges. This study addresses this issue by first employing pendant drop tensiometry and interfacial shear rheology to investigate the interface in the absence of external energy input.

The adsorption behavior of CRL at the octanol–water interface with varying pH is studied by measuring the interfacial tension (IFT) over time. Octanol is chosen as the oil phase due to the formation of extremely stable emulsions with small droplet sizes (5–10 μm).^36^ To assess the influence of charged nanoparticles at the interface on lipase adsorption, measurements were conducted both in the presence and absence of charged nanoparticles. By comparing individual experiments on enzyme adsorption, particle adsorption, and their simultaneous adsorption, we define a cooperativity value that quantifies the extent to which lipase adsorption is influenced by the presence of particles at the interface.

In the second part of this study, interfacial shear rheology is used to investigate the elastic properties of the interfacial layer constituted by enzymes and particles. Previous studies found that lipases as well as other proteins can form a skin-like interfacial layer at the oil/water interface, which is usually associated with unfolding and cross-linking of the proteins.^70,71^ Therefore, an increase in elastic modulus of the interface can be observed over time. Repeating measurements in the presence and absence of nanoparticles allows us to determine how the lipase layer interacts with adsorbed particles to establish an elastic interfacial network.

Since the composition of the interface may change when energy is introduced into the system, prepared w/o Pickering emulsions were analyzed using confocal laser scanning microscopy (CLSM) in the third part of this study. Fluorescent tagging enabled the visualization of CRL within the droplets. Subsequent image analysis allowed quantification of enzyme distribution as well as of the enzymatic layer thickness in biocatalytic PEs for the first time.

## Materials and Methods

### Chemicals

Lipase Type VII from *C. rugosa* was purchased from Sigma-Aldrich (Merck KGaA, Darmstadt, Germany). Disodium hydrogen phosphate dihydrate, sodium dihydrogen phosphate dihydrate, citric acid, trisodium citrate dihydrate were purchased from Carl Roth GmbH + Co.KG (Karlsruhe, Germany). 1-Octanol (99%) was purchased from Th.Geyer (Renningen, Germany). Analytical grade 1-octanol was purchased from Carlo Erba Reagents (Le Vaudreuil, France). Dimethyloctadecyl­[3-(trimethoxysilyl)­propyl]­ammonium chloride (60%) and octadecyltrimethoxysilane (90%) were obtained from Acros Organics (Geel, Belgium). Ethanol absolute (99.9%) was obtained from VWR chemicals BDH (Darmstadt, Germany). The fluorescent dye “Lightning red” was purchased from Serva (Heidelberg, Germany).

### Synthesis of Surface Modified Silica Particles

A detailed description of the procedure can be found in.^36,72^ In short, Ludox TM-40 particles (Sigma-Aldrich) were dialyzed for 10 days against roughly 50 L of deionized water using dialysis tubing with a molecular weight cutoff of 3500 Da. A suspension of about 1 wt % in ethanol was prepared. To obtain negatively charged particles (C18n−), the particles were silanized with octadecyltrimethoxysilane. The negative charge of the nanoparticles resulted from residual silanol groups, which are pH dependent (IEP between 2 and 3). For the positively charged surface modification (C18n+), dimethyloctadecyl­[3-(trimethoxysilyl)­propyl]­ammonium chloride was used instead. The positive charge was located at the quaternary amine and therefore not pH dependent. Afterward, the solvent was completely removed using a rotary evaporator.

The obtained particles were washed multiple times with Milli-Q water and once with a 30% ethanol solution. The washed particles were further purified through dialysis (molecular weight cutoff: 3500 Da) in absolute ethanol (99.9%) for 48 h. The particles were obtained as a dry powder. They are approximately 30 nm in size and partially hydrophobic (contact angle: 105–110°). The positively charged particles carry a charge of +53 mV and the negatively charged ones a charge of −50 mV in ethanol.^37^ They have a particle density of 2.15 g cm^–3^ and a specific surface area of 101 m^2^ g^–1^. Further details on the particle characterization can be found in previous studies by Stock et al.^37,73^

### Interfacial Tension Measurements

Interfacial tension measurements were performed with an OCA 25 (dataphysics-instruments, Filderstadt Germany) with the pendant drop method at 25 °C. For better handling and to save material, oil in water droplets were investigated. Thus, the needle tip had been bent to face upward and placed in a cuvette filled with the aqueous buffer phase. Buffers for pH 6–7 were prepared by mixing disodium hydrogen phosphate dihydrate and sodium dihydrogen phosphate dihydrate at a molar concentration of 100 mM. For pH < 6, citric acid and trisodium citrate dihydrate were mixed at the same concentration of 100 mM.

A buffer strength of 100 mM was used in this study as this concentration lies in the conventional range for biocatalysis. Enzyme solutions were freshly prepared by dissolving CRL in the buffer phase at 1 g/L (1.75 × 10^–5^ M) while gently stirring. Studies showed that this concentration is sufficient to achieve a saturated interface.^61^ The syringe was filled with 1-octanol, which was saturated with the respective buffer before. To test the purity and cleanliness of the system, the device was calibrated with a drop of 1-octanol in deionized water at IFT values of 8–8.2 mN/m prior to each measurement. This was in agreement with literature data.^74^

The drop volume was maintained at a constant value of 5.5 μL $\left(\approx 15mm^{2}\right)$ . For particle adsorption, particles were dispersed in 1-octanol in a sonic bath for at least 20 min at a weight fraction of 0.5 wt %. With a specific particle cross section of 27.6 m^2^ g^–1^ ^37^ this concentration is sufficient to cover the interface and to avoid particle depletion inside the droplet. Each measurement was repeated at least three times.

### Interfacial Shear Rheology

The interfacial viscoelastic behavior was studied by performing oscillatory shear deformation using the MCR 302 (Anton Paar, Graz Austria) rheometer and an interfacial rheology cell with a biconical disk (radius: 34.14 mm; angle: 5°). The cell was maintained at a temperature of 25 °C. First, the aqueous phase was added and the biconical disk was lowered to the surface. Afterward, 1-octanol was carefully added on top. Both phases had approximately the same amount of liquid. By applying a defined oscillatory strain

1 $$\gamma \left(t\right)=\gamma_{A}\cdot \sin\left(\omega t\right)$$

on the interface, a stress response

2 $$\tau \left(t\right)=\tau_{A}\cdot \sin\left(\omega t+\delta \right)$$

with a specific phase angle δ is measured. This phase angle corresponds to the viscoelastic behavior of the interface. A purely elastic response correlates to a phase angle of 0° and a purely viscous surface shows a phase angle of 90°. Between 0° and 90°, the behavior is viscoelastic and can be used to calculate the interfacial storage *G*_i_^′^ and loss modulus *G*_i_^″^ using eqs 3 and 4.

3 $$G_{i}^{\prime }=\frac{\gamma_{A}}{\tau_{A}}\cos\left(\delta \right)$$

4 $$G_{i}^{\prime \prime }=\frac{\gamma_{A}}{\tau_{A}}\sin\left(\delta \right)$$

The buildup of the interfacial layer was monitored by applying a constant strain of γ = 0.1% and a angular frequency of ω = 1 rad/s. To test the resistance of the interfacial layer against deformation, amplitude sweeps were performed by gradually increasing the applied strain from 0.01 to 100% with a constant angular frequency of 1 rad/s.

### Preparation of Fluorescent w/o Pickering Emulsions

Enzymes were fluorescently labeled by mixing 1 mL of a 0.5 g/L CRL solution (phosphate buffer) with 2.53 μL of lightning red dissolved in anhydrous DMSO at pH 8.3. This is the amount needed for each molecule to be labeled once. To ensure the reaction was fully completed before preparing the PE, the solution was incubated for 30 min. As the reaction progressed, a shift in color was observed. The respective nanoparticles were dispersed in 1-octanol in a sonic bath for at least 20 min with a weight fraction of 0.5 wt %. Water-in-oil Pickering emulsions were prepared by dispersing 1-octanol together with the labeled enzyme solution at an aqueous phase volume fraction of 0.2 with an IKA T 25 digital UltraTurrax S25N-18G at 25,000 min^–1^ for 2 min. The obtained emulsions are stable against coalescence for at least several weeks.

### Confocal Laser Scanning Miscrosopy

Droplets of fluorescent PEs were placed on a microscope coverslip and imaged with a Luminosa confocal laser scanning microscope (PicoQuant, Berlin Germany). A picosecond pulsed excitation laser with a wavelength of 530 nm (LDH-P-FA-530L) was set to a constant excitation power of 30 μW and a pulse repetition rate of 32 MHz. The sample was imaged with a 60× water immersion objective with NA 1.2 (Olympus, Tokyo Japan). The pixel size was set to 100 nm. An emission bandpass filter of 600/50 nm was placed in front of the SPAD detector (Excelitas, Pittsburgh USA). The arrival times of single photons were registered with a MultiHarp 150 TCSPC module (PicoQuant, Berlin Germany).

### Image Analysis

Images taken with the CLSM were analyzed using a custom script in ImageJ. To obtain a full radial intensity profile, a line profile along the droplet diameter was rotated by 1° increments through 360° around the droplet center. All line profiles were exported to Python for further processing. The intensity curves were averaged to obtain a representative enzyme concentration profile across the droplet. From this profile, several parameters were extracted: droplet size, enzyme concentration at the intensity peak (i.e., at the interface), enzyme concentration in the bulk phase of the droplet, and the interface thickness. The latter was defined as the intensity peak, corresponding to the initial enzyme concentration of 0.5 g/L.

## Results and Discussion

### Adsorption at the o/w-Interface

#### CRL Adsorption

Interfacial tension of the octanol/water interface in the presence of CRL was measured for different pH values via the pendant drop method (see Figure 2a). With CRL adsorption at the o/w interface, the IFT is expected to decrease proportionally, providing direct information on the adsorption rate. However, strictly speaking, the Young–Laplace equation, used in the pendant drop method, is not entirely valid for elastic interfaces. The following IFT values can therefore be regarded as an “effective” interfacial tension. Measurements were performed at a fixed enzyme concentration of 1 g/L, as interfacial saturation had been reached under these conditions (see Figure S1). The associated surface pressure^75^

5 $$\Pi =\text{IF}T_{o/w}-IFT$$

close to saturation (averaged decline of the last 3 min $<0.005\frac{mN/m}{\min}$ ) is depicted in Figure 2b. At reduced electrostatic repulsion between proteins, near the isoelectric point (IEP), higher packing at the interface results in increased surface pressure. Higher surface pressures around the IEP have been reported for other proteins, such as β-lactoglobulin,^76,77^ bovine serum albumin (BSA),^77,78^ catalase, and lysozyme.^79^

**Figure 2:**
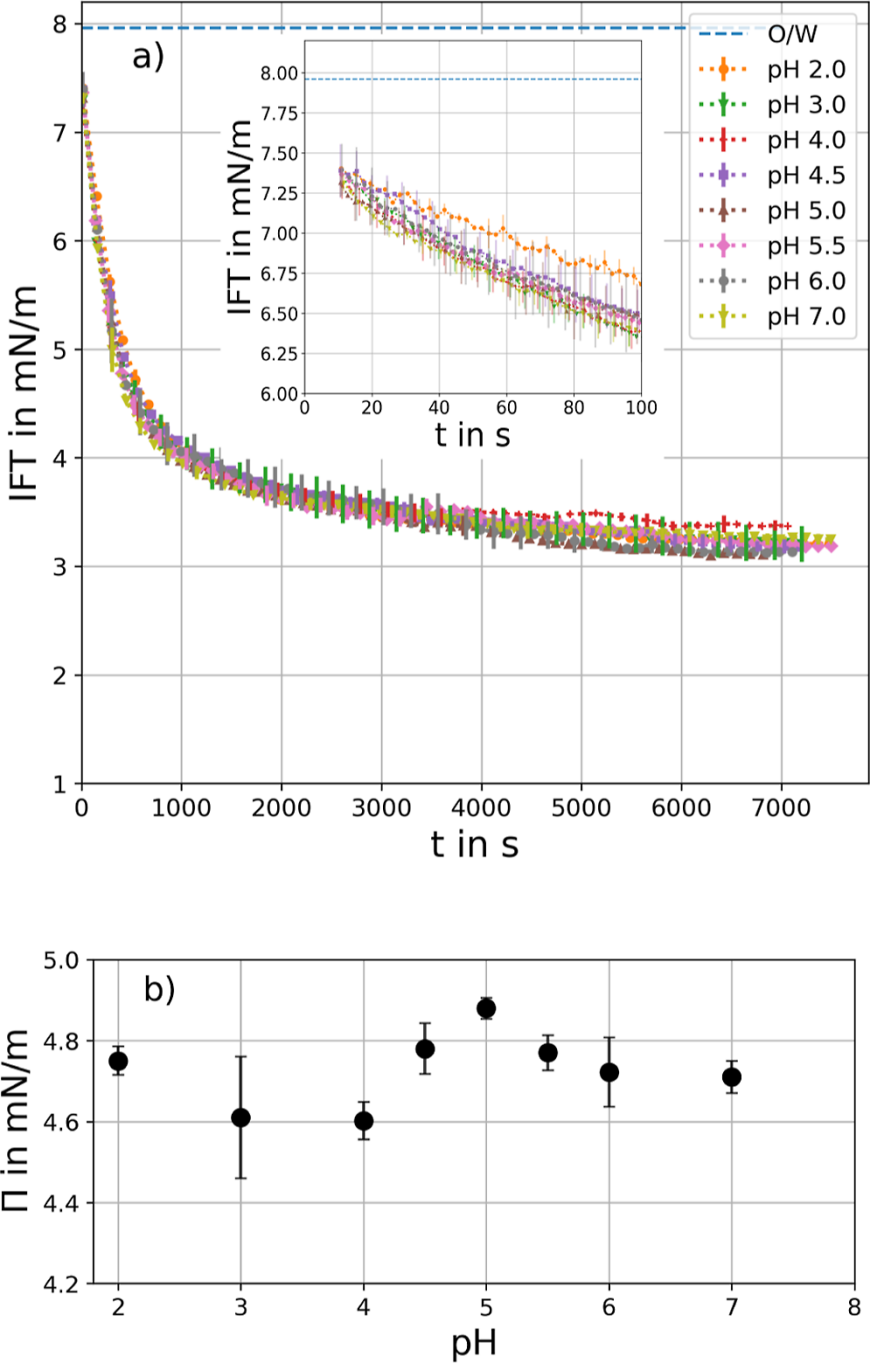
(a) Interfacial tension (IFT) of the 1-octanol/buffer interface during CRL adsorption for different pH values. Enzyme concentration was 1 g/L for all measurements. Error bars are shown every second point for better visibility and show standard deviation of three measurements. (b) Surface pressure Π = IFT_o/w_ – IFT at *t* = 7000 s. All measurements were taken three times.

In this study, a slight increase in surface pressure was observed at the IEP of CRL (5.0 ^36^). To the best of our knowledge, there is no literature data that shows the influence of pH on lipase adsorption onto a liquid–liquid interface. The relatively small change in surface pressure for CRL near the IEP could be attributed to the high buffer concentration of 100 mM, which screens the electrostatic interactions between proteins through counterions in pH regions far from the IEP. The IFT for the pure buffer/octanol-interface remained constant for different pH values (see Figure S2).

#### Adsorption of Particles

As mentioned above, solid particles in the submicron scale adsorb strongly at liquid/fluid interfaces as they decrease the total energy of the system. Due to the high desorption energy, which can be calculated for spherical particles at the o/w-interface according to eq 6

6 $$E=\pi r^{2}\text{IF}T_{o/w}{\left(1-\theta \right)}^{2}$$

where *r* is the radius of the particle, blue IFT_o/w_ is the interfacial tension of the L/L-interface and θ is the three-phase contact angle, adsorbed nanoparticles are said to be irreversibly attached to the interface.^80^ However, as for the case of charged colloid particles, repulsive interactions between charged particles and the charged liquid surface may hinder spontaneous adsorption even against favorable adsorption energy.^46^ By measuring the electrophoretic mobility of oil droplets, experimental studies have established the negative zeta potential of the oil/water interface,^81^ which increases to more negative values while pH is increased.^82,83^

Similar conclusions were derived for the air/water interface from thin film pressure balance measurements at wetting films^84^ and foam films.^85^ Yet the explanation for this observation is still a matter of debate.^82,83,86^ A common explanation is the specific adsorption of hydroxide ions at the interface.^83,87^ Water molecules are highly ordered in the presence of an apolar liquid, with the oxygen atom oriented toward the hydrophobic surface.^88^ This orientation supports selective hydroxide adsorption through the formation of strong dipoles or hydrogen bonds between the ordered water molecules and the adsorbed hydroxide ions.^81^ Through sum frequency scattering at the oil/water interface, Vácha et al. observed no evidence of a selective adsorption of hydroxide ions. From molecular dynamics simulations they concluded that the water molecules close to the interface become partially charged which results in a net charge oscillation in the proximity of the interface.^82^

With this information in mind, the results obtained in this study are in agreement with the aforementioned work. The results for nanoparticle adsorption are depicted in Figure 3. During the adsorption of positively charged particles at the octanol/water interface (Figure 3a), a decrease in IFT is observed. As pH increases, the surface pressure induced by the nanoparticles also rises. Since the positive particle charge remains unchanged with pH due to the quaternary amine, this effect is attributed to the increasingly charged interface at higher pH. The adsorption kinetics follow a similar pattern, with an initial rapid drop followed by a slower decline over time.

**Figure 3:**
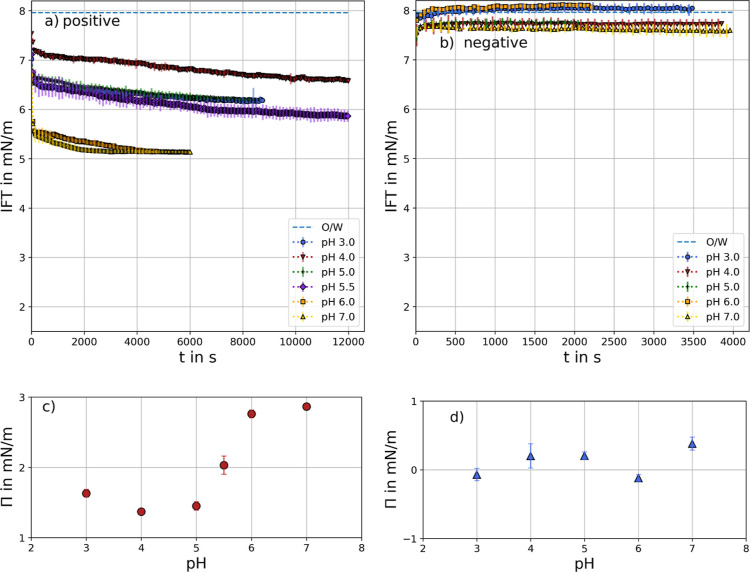
Interfacial tension of (a) positively charged particles or (b) negatively charged particles adsorbing at different pH values. 0.5 wt % particles were dispersed in 1-octanol and measured against buffer solutions of different pH values. Error bars are shown every second data point for better visibility and depict standard deviation of three measurements. Surface pressure Π calculated at steady state conditions for (c) positively charged particles (red circles) and for (d) negatively charged particles at *t* = 2000 s (blue triangles).

A Similar behavior for positively charged silica particles dispersed in the aqueous phase was observed at the o/w interface with crude oil.^89^ Nanoparticle adsorption at the interface was primarily driven by the presence of polar carboxylic acid groups, which become negatively charged at higher pH and promote electrostatic attraction to the particles. To confirm that the observations in this study were not influenced by impurities in the organic phase, control measurements were conducted using analytical-grade 1-octanol, showing no detectable differences (see Figure S3).

In contrast, the negatively charged particles (Figure 3b) do not decrease the IFT or just have a negligible influence. Furthermore, a significant trend with increasing pH observed is not observed, as the surface pressure fluctuates around zero. The question now arises whether negatively charged particles spontaneously adsorb at the interface. As mentioned earlier, there is no clear consensus on whether IFT measurements definitively indicate particle adsorption or, in other words, whether IFT changes upon particle adsorption. Fan and Striolo argue that the effect of adsorbed particles on IFT depends on surface coverage and particle–particle interactions. They suggested that experiments showing no change in IFT may lack the necessary surface density and particle–particle interactions to produce a measurable effect. In addition, they proposed that repulsive particle–particle interaction leads to reductions in IFT, while attractive interactions will slightly increase the IFT.^47^

Following this argumentation, it could be concluded that the provided surface coverage was not enough to show any significant results for the negatively charged particles, although the same concentration was sufficient for the positively charged particles to influence the IFT. Further increasing the weight percentage of particles to 2 wt % did not result in a stable dispersion. In contrast to the positively charged particles, there seems to be a slight increase in IFT in the first few minutes after droplet formation before a plateau is reached. This could indicate weak particle–particle interactions or adsorption of small impurities.

Additional adsorption measurements were performed using unmodified Ludox particles dispersed in the aqueous phase under identical conditions to validate the findings obtained with the negatively charged particles. The resulting surface pressure exhibited similar fluctuations around zero, consistent with those observed for the modified particles (see Figure S4). Interfacial shear rheology can yield further indication of potential adsorption of negatively charged particles.

#### Simultaneous Enzyme-Particle Adsorption at the L/L-Interface

After analyzing CRL and particle adsorption separately, the next step is to study their simultaneous adsorption, as this scenario best reflects practical applications. The results are depicted in Figure 4. The initial drop in IFT becomes more pronounced with increasing pH, suggesting accelerated adsorption in the presence of positively charged particles (see Figure 4a). Subsequently, pH 3 to 5.5 follow a similar trend, while the decline in IFT is more pronounced at pH 6 and 7.

**Figure 4:**
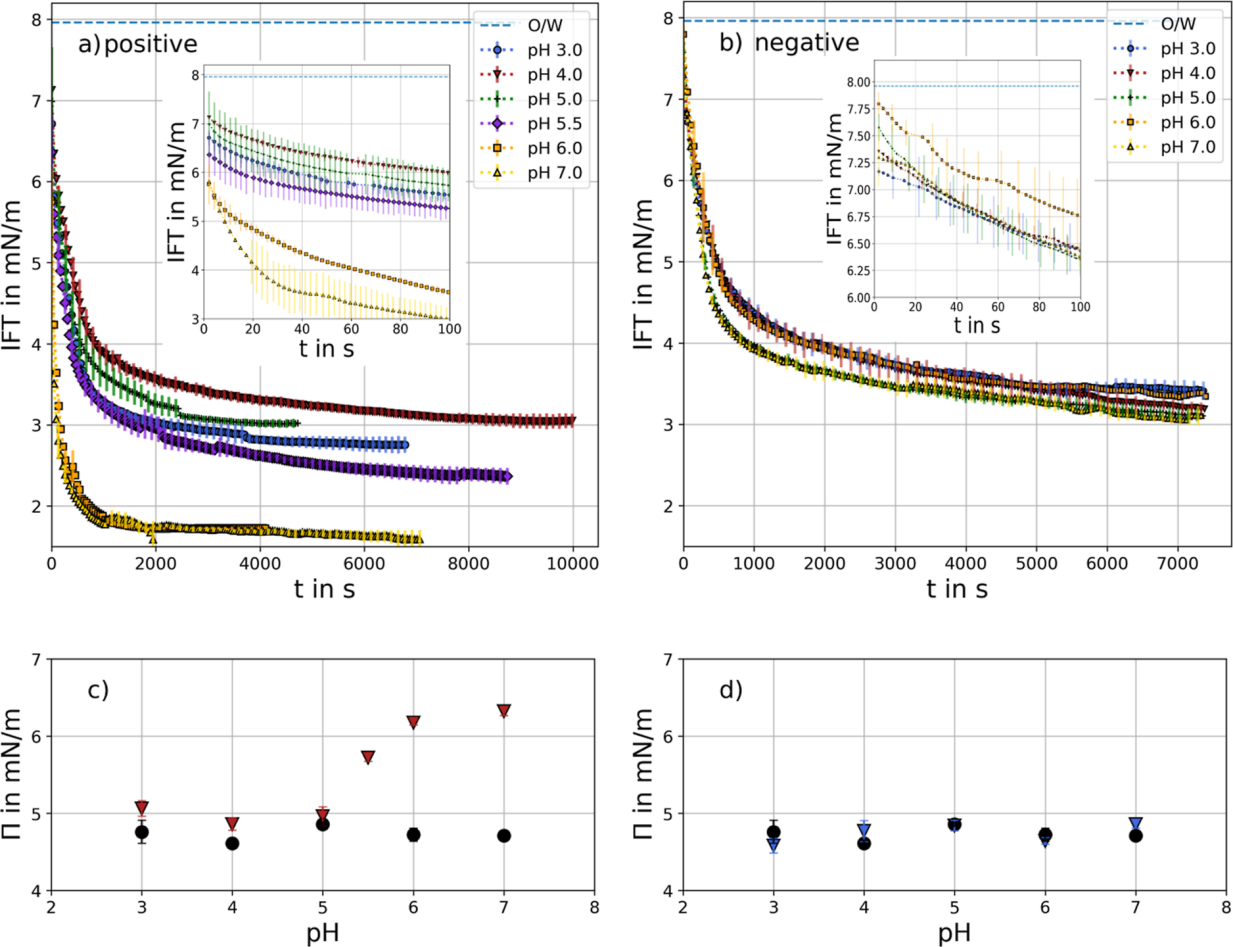
Simultaneous adsorption of CRL and (a) positively charged particles or (b) negatively charged particles at different pH values. Enzyme solutions were prepared with 1 g/L. 0.5 wt % particles were dispersed in 1-octanol. Error bars are shown every third data point for better visibility and depict standard deviation of three measurements. Surface pressure Π for CRL and (c) positively charged particles (red triangles) and for (d) negatively charged particles (blue triangles) at steady state conditions. For comparison, the respective interfacial pressure of CRL is marked with black circles.

Furthermore, surface pressure at stationary conditions reach higher values for pH above 5.5 (see Figure 4c). This can be explained by an additive effect of the two stabilizers: enzymes and nanoparticles. Electrostatic attraction between the positively charged particles and the negatively charged enzymes (with an IEP at pH 5) increases the interfacial pressure, particularly at higher pH values. In contrast, the adsorption of CRL and negatively charged particles does not show any significant difference in surface pressure compared to the sole CRL adsorption.

To estimate the influence of particles at the interface on lipase adsorption, we define a cooperativity factor, α, as shown in eq 7, by relating individual to simultaneous adsorption. To isolate the enzyme’s contribution to the interfacial tension (IFT) during simultaneous adsorption, the surface pressure from particle adsorption alone (Π_particles_) is subtracted from the total surface pressure during simultaneous adsorption (Π_sim_). This difference is then normalized by the surface pressure from enzyme adsorption alone. A cooperativity factor of 1 indicates that lipases and nanoparticles adsorb independently, whereas a value below 1 suggests interactions between the particles and enzymes, implying that the interface is partially blocked by the particles.

7 $$\alpha =\frac{\Pi_{sim}-\Pi_{particle}}{\Pi_{enzyme}}$$

The results of the cooperativity factor α are shown in Figure 5. The simultaneous adsorption of CRL in the presence of negatively charged particles is comparable to the sole adsorption of CRL (see Figure 4d), and the influence of negatively charged particles on the IFT was relatively low. Consequently, enzyme adsorption is not influenced by the presence of negatively charged particles in the oil phase, leading to complete enzyme surface coverage and an α of 1.

**Figure 5:**
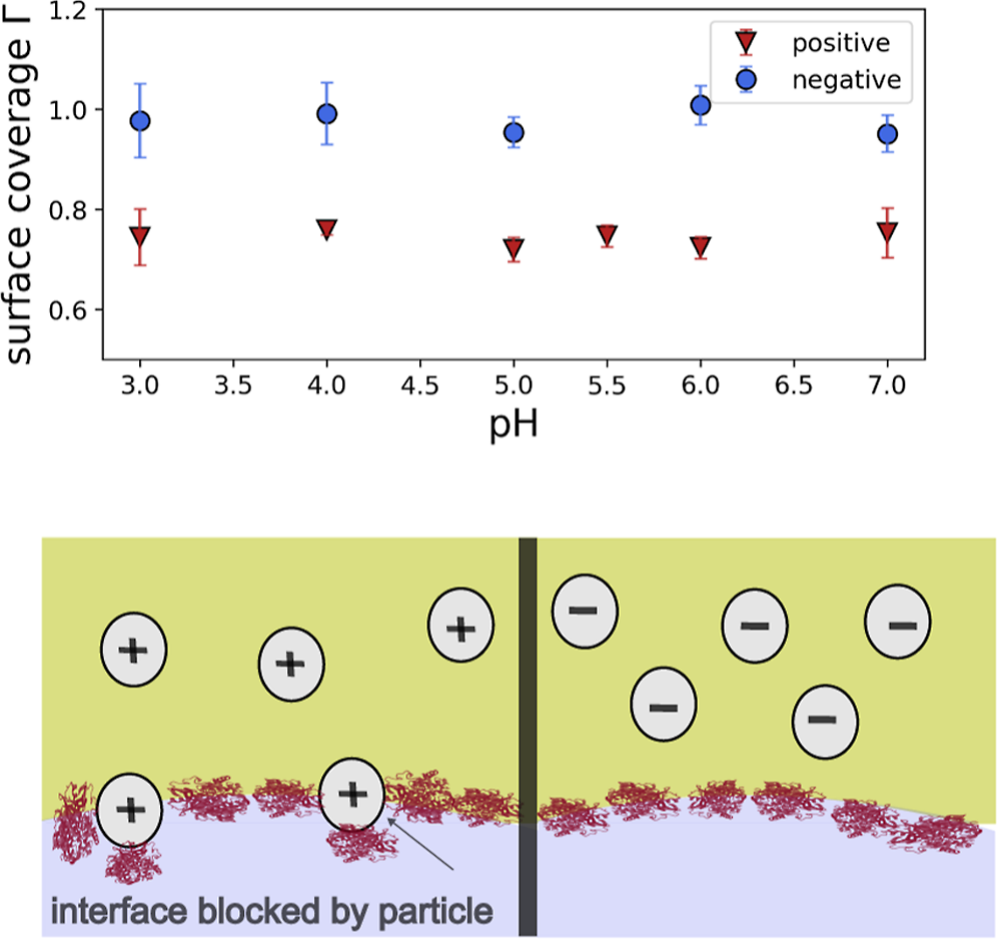
Top: Cooperativity α according to eq 7. Bottom: envisaged scheme of the simultaneous interface coverage by enzymes and particles.

In contrast, the cooperativity α in the presence of positively charged particles is reduced to roughly 0.75. This suggests an interaction between CRL and particles. Figure 5 illustrates this effect, showing how particles occupy adsorption sites and prevent enzymes access to the interface, thereby reducing α. Surprisingly, this effect is constant with pH. As positively charged particles exhibit lower surface pressure at lower pH (see Figure 3a), IFT results from simultaneous adsorption is similarly reduced in the acidic region. To further confirm the proposed interaction between the positively charged particles and CRL at the interface, interfacial shear rheology tests were performed.

### Interfacial Shear Rheology

#### Influence of Lipases

The viscoelasticity of the interfacial CRL layer at the octanol/water interface was studied for pH 3 and 7. From Figure 6a, a slow buildup of an elastic interfacial layer over several hours can be observed. It is known that lipase molecules form a skin-like surface layer in the course of a few hours. This behavior is attributed to denaturation of the enzyme at the interface and possible formation of cross-links via disulfide-bridges.^70^ This is additionally emphasized by the 3-fold increase of the elastic modulus *G*_i_^′^ when lowering the pH from 7 to 3, where CRL most likely has a looser and less globular structure, which then supports intermolecular interaction.

**Figure 6:**
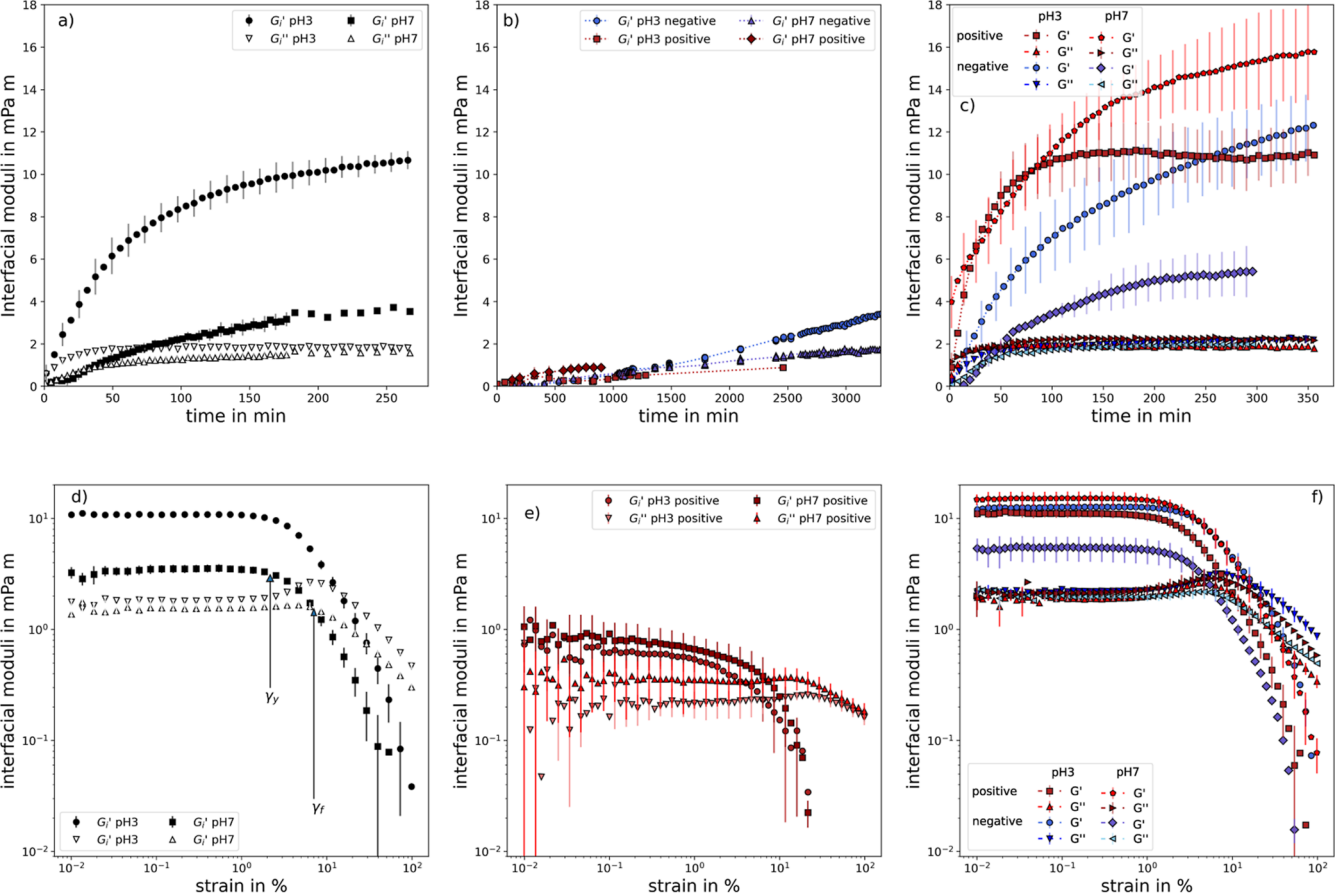
Interfacial loss *G*_i_^″^ and storage modulus *G*_i_^″^ as a function of time (a–c) of the CRL layer (a), particle layer (b) and CRL-particle network (c) on the octanol/water-interface for pH 3 and 7, respectively. Time tests were performed with a constant strain of 0.1% and an angular frequency of 1 rad/s. Enzyme solutions were prepared with 1 g/L and 100 mM buffer. Particles were dispersed in 1-octanol at 0.5 wt %. Amplitude sweeps (d–f) of the CRL layer (d), particle layer (e) and CRL-particle network (f) were performed with a constant angular frequency of 1 rad/s after buildup of the respective layer. Amplitude sweeps for particles were performed only with positively charged particles, since the slope of storage modulus over time for the negatively charged particles did not decline even after 58 h and particles visibly sedimentated after 40 h. Error bars are shown every second point for better visibility and depict the standard deviation of three measurements.

Furthermore, the amplitude sweep in Figure 6d provides information about the resistance to shear deformation of the interfacial layer. By systematically increasing the deformation amplitude, the elastic structures at the interface start to loosen and finally break. While the yield point (γ_y_), which marks the onset of structural weakening, remains largely unchanged between the two pH values, the flow point (γ_f_), indicating the transition from gel-like to fluid behavior, increases from pH 7 to 3. At this point, the intermolecular cross-links, which are more pronounced at pH 3, begin to break.

#### Influence of Particles

Figure 6b,e shows the results of interfacial oscillating shear measurements on the octanol/water interface with particles dispersed in the octanol phase. It took the particles a significantly longer time than CRL to form a measurable elastic response through adsorption at the interface. On the other hand changes in IFT could be seen almost immediately (see Figure 3a). One possible explanation could simply lie in the pendant drop technique itself. The particle interface coverage must be much higher to form an elastic surface layer than to induce changes in the IFT. The isothermal time test in Figure 6b shows that the elastic modulus of positively charged particles at pH 7 reaches saturation after 700 min, consistent with other studies on nanoparticles.^90^

However, this process is significantly slower and results in a lower storage modulus compared to CRL. At pH 3, the storage modulus (*G*_i_^′^) is even lower due to the less charged o/w interface, which attracts the positively charged particles. In comparison, Alsmaeil et al. measured the dilatational modulus of positively charged particles, previously dispersed in the aqueous phase, at the o/w interface using the oscillating drop method. They observed a significant increase in the modulus at pH 9, consistent with their IFT measurements.^89^ This pronounced increase, however, did not show in the present study, possibly due to differences between the two measurement techniques.

Evaluating the negatively charged particles is more challenging, as *G*_i_^′^ continues to increase over time without reaching saturation, even after 58 h of measurement. This behavior suggests particle sedimentation, which was visually confirmed by slight clouding at the interface after approximately 24 h (see Figure S5). Overall, while the adsorption of negatively charged particles at the o/w interface cannot be entirely ruled out, IFT results suggest that these particles most likely remain suspended above the interface due to sedimentation rather than actual adsorption. Consequently, amplitude sweeps were conducted only for positively charged particles after 20 h. However, these results should also be interpreted with caution, as signs of sedimentation were observed, albeit at a slower rate. A slight clouding near the interface became visible after 48 h.

A more detailed analysis of particle sedimentation behavior in octanol is provided in the Supporting Information. At higher water content in octanol, electrostatic repulsion is reduced due to screening effects, leading to particle sedimentation (see Figures S6 and S7). This effect is more pronounced for negatively charged particles and appears to be influenced by the container material, as similar sedimentation behavior could not be reproduced in plastic tubes (see Figure S7). In the context of interfacial shear rheology, the results are not expected to be affected within the measurement time frame, as dried octanol (0.03–0.06 wt %) was used. For water to have a significant influence, it must diffuse into the octanol layer, and even after 24 h, the bulk particle concentration is reduced by only about 20%.

#### Influence of Simultaneous Lipase-Particle Adsorption

Viscoelastic measurements were conducted at the octanol/water interface with CRL in the aqueous phase and particles in the organic phase. In Figure 6c, the increase of the elastic modulus over time closely resembles that of the pure CRL adsorption process. This is expected, as the diffusion and adsorption of particles at the interface occur over a much longer time frame compared to CRL. Nonetheless, the presence of charged particles in the octanol phase can significantly influence the adsorption process and the resulting elasticity of the interface.

Elasticity values in the linear viscoelastic (LVE) range are summarized for all experiments in Figure 7a. At pH 7, the interfacial elasticity increases approximately 4-fold in the presence of positively charged particles. IFT measurements proposed indicated cooperative interaction between the particles and enzymes through spontaneous adsorption. This further supports the proposed enzyme–particle network indicated by simultaneous adsorption. Once adsorbed, the particles remain strongly attached to the interface and likely enhance the network structure through electrostatic attraction with the enzyme. This interaction reinforces protein entanglement and cross-linking, resulting in a highly shear–resistant interface, as illustrated in Figure 8a.

**Figure 7:**
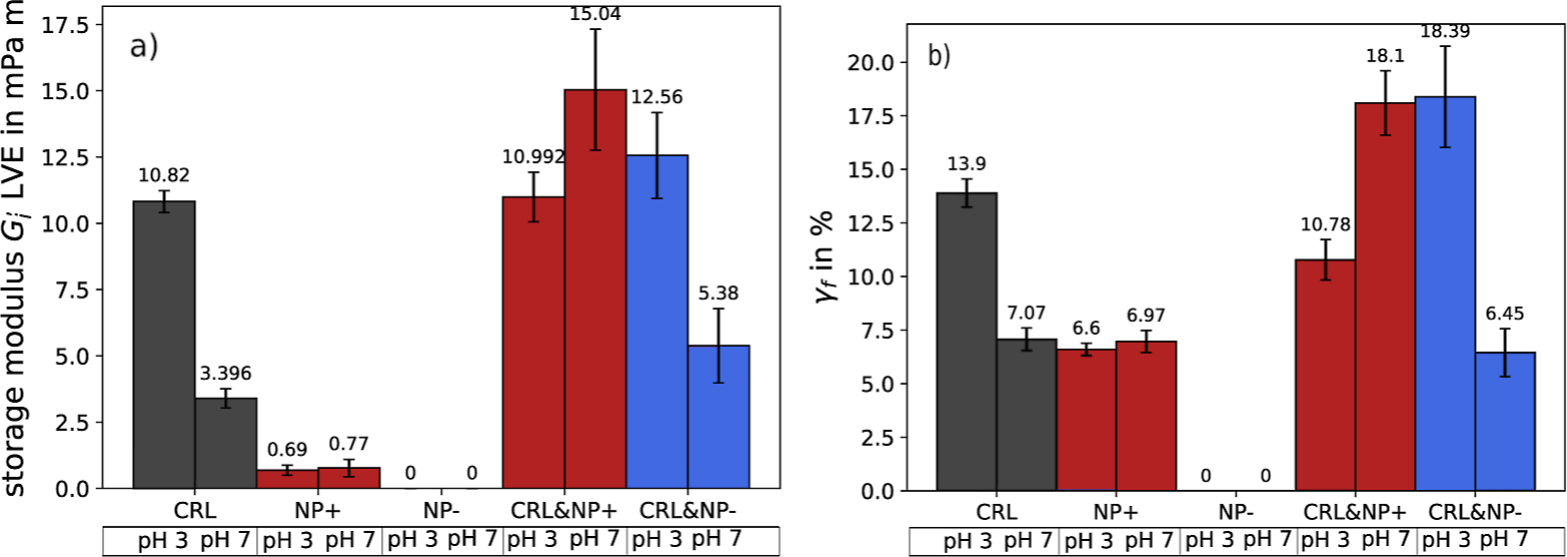
Summary of the interfacial storage modulus (a) in the LVE range and the flow point (b). The flow point was determined as the intersection of the loss and storage modulus by linear approximation.

**Figure 8:**
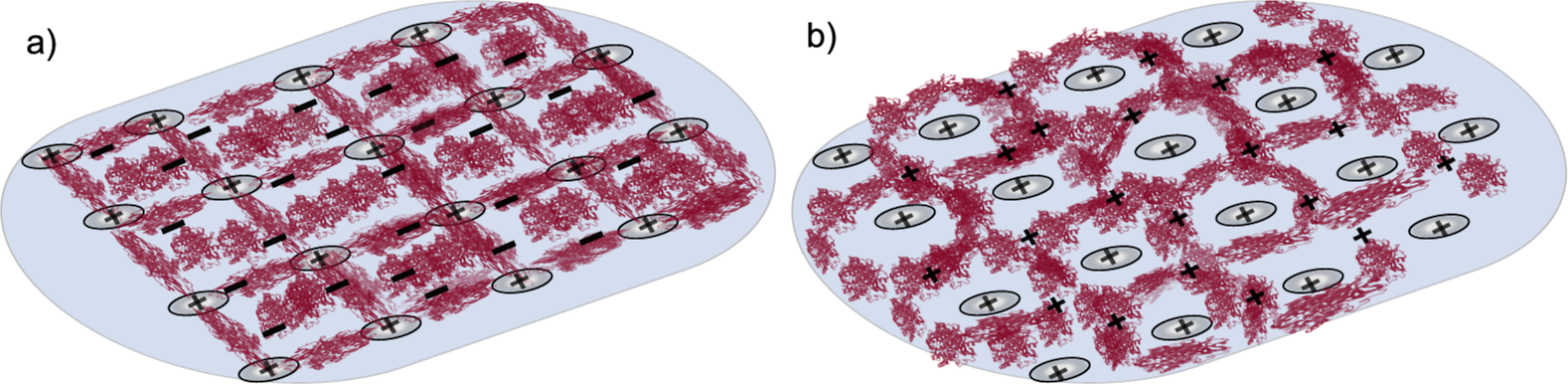
Suggested structure of the o/w interface with positively charged particles and CRL. At pH 7 (a) CRL contains a negative net charge and a network structure with the nanoparticles could form. At pH 3 (b), both CRL and NPs are positively charged and would repel each other and only intermolecular links between CRL can form.

At pH 3, the storage modulus for simultaneous adsorption remains unchanged compared to enzyme adsorption alone. Although particles may occupy part of the o/w interface—as indicated by IFT and elasticity data—repulsive interactions between particles and enzymes hinder the formation of an additional network that could enhance interfacial elasticity, as illustrated in Figure 8b.

As for the negatively charged particles, only a slight increase can be observed at pH 7. Although they do not adsorb at the interface, their proximity seems to influence the measured elasticity for simultaneous adsorption. The same increase of about 2 *mPa*·*m* happens at pH 3. At this stage, further research is required to explain this behavior. It is also possible that this effect occurs because the measuring system cannot resolve the interface on a molecular level and instead detects particle layers above the interface. But in comparison to the strong increase caused by positively charged particles, this effect does not appear significant.

Further insights into the brittleness or stiffness of the interfacial layer can be obtained from the yield and flow point.^91^ The yield point γ_y_ remains relatively unchanged, with an average at a strain of 1.65% (excluding the particle measurements). A clearer picture emerges when examining the flow point, as shown in Figure 7b. The higher the flow point, the greater the resistance to complete destruction of the interfacial network structure. Overall, the flow point is correlated with elasticity, meaning that a higher value in the storage modulus results in a later flow point.

One exception is the case of simultaneous adsorption of enzymes and positively charged particles at pH 3. The flow point decreases from 13.9% (in the case of sole enzyme adsorption without particles) to 10.78%, while the interfacial storage modulus remains constant. Compared to the surface covered only by CRL, part of the surface is blocked by particles, which impose electrostatic repulsion on the enzymes (see Figure 8b). For the intertwined CRL network covering the interface, these particles create small gaps that disrupt the overall integrity of the layer. At small deformations in the LVE range, this does not affect the storage modulus, but at higher strains, the structural layer becomes more brittle.

### Interfacial Layer Thickness in Pickering Emulsions

Lastly, we investigated the enzyme-particle-layer at the interface after intensive energy input and preparation of the PE. While previous experiments focused on the interface without external energy input, Pickering emulsions are typically prepared using vigorous stirring or sonication to overcome energy barriers and promote particle adsorption at the interface. If the applied force exceeds the electrostatic repulsion between the particle and the interface, particle adsorption becomes favorable,^92^ leading to the formation of stable Pickering emulsions even for negatively charged particles. Since energy input determines the resulting droplet size, we selected a rotational speed above the threshold where droplet size remains constant.^69^

High-resolution images of droplets containing fluorescently labeled enzymes were captured using a CLSM, and their fluorescence intensity was analyzed. In Figure 9a, the intensity of each pixel is plotted across the diameter of the droplet and the averaged results are summarized in Table 1. The labeled enzymes are initially located in the aqueous phase before emulsification and adsorb at the nascent ”inner” interface during (or prior to) the emulsification process. This is reflected in the peak asymmetry observed in the intensity plot in Figure 9a. The signal decays more sharply on the oil-facing (outer) side of the droplet than on the aqueous (inner) side. Additionally, while the intensity reaches zero on the outside, it remains above zero in the droplet center.

**Figure 9:**
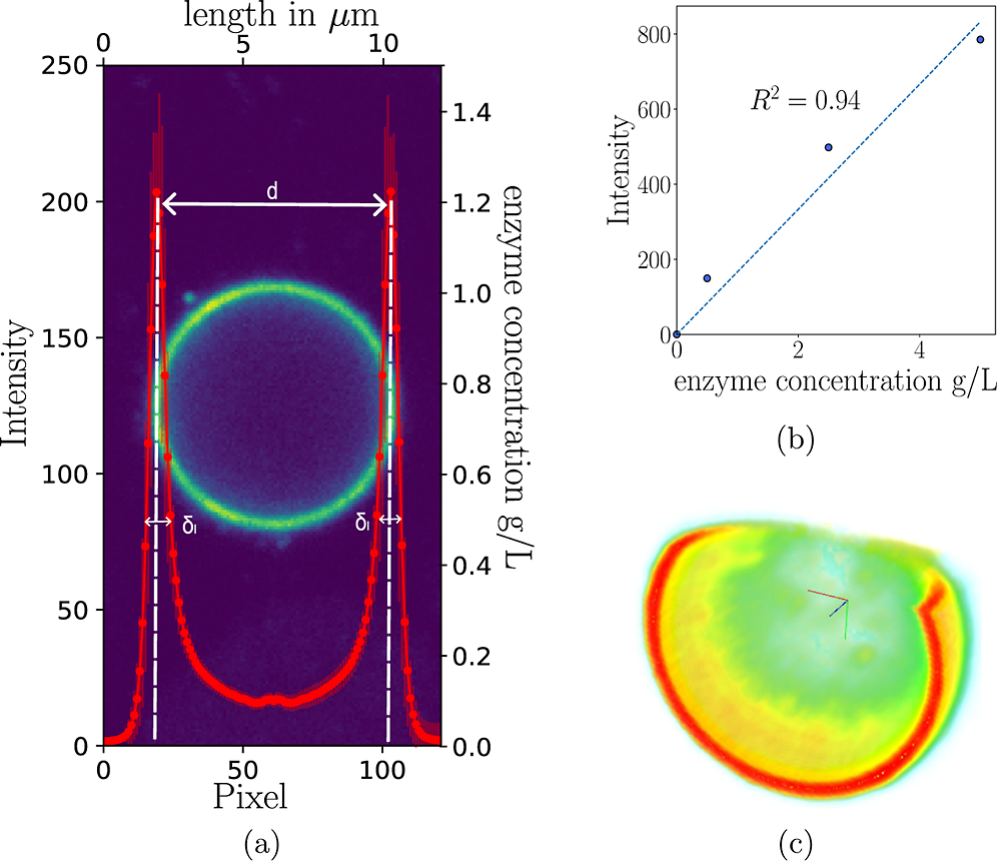
(a) Background: selected fluorescence intensity image of a representative w/o droplet. Red: corresponding averaged intensity plot. PE prepared with 0.5 wt % negatively charged particles, 0.5 g/L CRL in buffer at pH 8.3. (b) Calibration curve. (c) Cross section of a 3D-rendered z-stack of a droplet. Color scheme represents intensity values (high intensity:red, low intensity:green). Droplet diameter: 6 μm.

**Table 1:** Results of the High-Resolution Image Analysis for Both Particle Types^a^

	NP+	NP-	no particles
droplet diameter μm	5.17 ± 1.78	3.86 ± 1.23	
interface conc.^b^ g/L	1.56 ± 0.41	1.2 ± 0.35	1.27 ± 0.43
interface thickness^c^ μm	0.75 ± 0.2	0.51 ± 0.19	1.48 ± 0.13
bulk conc.^d^ g/L	0.098 ± 0.09	0.097 ± 0.09	

a More than 70 droplets were analysed for the negatively charged and 140 for the positively charged particles. PEs prepared with 0.5 wt % particles with 0.5 g/L labeled enzyme solution at pH 8.3.

b Concentration measured at peak.

c Width at constant peak height corresponding to 0.5 g/L.

d Average concentration of 10 pixels around the droplet’s center.

To ensure that the observed fluorescence emission originates solely from the labeled enzyme, control samples containing unlabeled enzyme in buffer, dispersed particles in octanol, and PEs with unlabeled enzymes were also imaged. While the unlabeled enzyme remained undetectable, the nanoparticles exhibited slight autofluorescence (see Figure S8). However, their signal intensity was only approximately 5% of that from the fluorescently labeled enzyme, making a significant influence on the results unlikely. For comparison, the interfacial layer thickness of CRL was also measured in the absence of particles. As CRL alone is not able to stabilize the emulsions, a droplet of fluorescently labeled enzyme solution was placed on a microscope slide and covered with octanol (see Figure S9).

From the distribution shown in Figure 9a, it is clear that labeled enymes accumulate at the interface, forming multilayers of approximately 0.75 μm for positively charged particles and 0.51 μm for negatively charged particles, respectively. Compared to the enzyme layer formed through spontaneous adsorption, the formation of a Pickering emulsion results in a thinner interfacial layer for both particle types. At pH 8.3, electrostatic attraction between CRL and positively charged particles leads to a comparatively thicker interfacial layer with higher enzyme concentration and packing density. In contrast, repulsive interactions with negatively charged particles result in lower interfacial concentrations, resembling those observed under spontaneous adsorption.

This is in agreement to the representation of the interface proposed earlier (Figure 8a). However, this effect could potentially be influenced by droplet size, as the PE prepared with positively charged particles exhibited a slightly larger droplet diameter. Although Figure S10 shows no clear correlation between interface concentration and droplet diameter, the average bulk concentration as well as the layer thickness remains consistent for both. Since the same amount of enzyme was used in both PEs, the difference in the amount of enzyme that cannot accumulate at the interface is available for the formation of another droplet. This results in greater number of smaller droplets. To confirm this causality, a more systematic study of the interface at different droplet sizes would be necessary.

Additionally, it is important to note that labeled CRL exhibits slightly different adsorption behavior compared to unlabeled enzyme within a PE. Attachment of the fluorescent dye reduces the surface activity of CRL to approximately 62% of the surface pressure observed for unlabeled CRL under identical conditions (Figure S11). This suggests that the method likely underestimates the packing density, as unlabeled lipases exhibit stronger adsorption in the absence of the fluorescent label. At the same time, it may overestimate the interfacial layer thickness due to the increased molecular size caused by the dye attachment.

## Conclusions

Previous studies of biocatalytic PEs regarded the droplets as a “black box”, with the exact location of the enzyme and the influence of pH and nanoparticle charge remained largely unknown. This study investigated the interfacial structure of the CRL layer in the presence of positively or negatively charged silica particles at different pH values and a buffer strength of 100 mM at the octanol/water interface using a combination of complementary techniques.

The pendant drop technique was used to examine the simultaneous adsorption of CRL and nanoparticles at the o/w interface, investigating potential competitive or cooperative interactions. Our findings indicate that positively charged silica particles reduce the IFT by adsorbing at the interface, which becomes more pronounced as pH increases and the interface becomes more negatively charged. In contrast, negatively charged particles had no impact on the IFT, suggesting that they do not spontaneously adsorb at the o/w interface, whereas positively charged particles do.

Investigation of the simultaneous adsorption of nanoparticles and CRL hints at a cooperative network formation for positively charged particles at the interface. Both species adsorb simultaneously to reach IFT values below individual adsorption. The relation between individual and simultaneous adsorption, represented by the factor α, reached a constant value of 0.75 for the whole pH range. In contrast, negatively charged particles did not affect the adsorption process of CRL.

Oscillatory shear measurements at the interface further confirmed the formation of an elastic network due to electrostatic attraction between positively charged nanoparticles and CRL. At pH 7, attractive interactions between the positively charged particles and negatively charged enzymes led to a significant increase in the interfacial storage modulus. In contrast, repulsive interactions at pH 3 resulted in reduced interfacial elasticity. Consistent with the adsorption results, negatively charged particles had no significant effect on interfacial elasticity, suggesting that they do not adsorb spontaneously.

Introducing energy into the system led to the formation of a stable w/o Pickering emulsion for both particle types. Fluorescent labeling of CRL enabled the visualization of enzyme distribution inside the droplets using CLSM. This method bridges the insights gained from the self-assembled interface in the first two techniques with the interfacial structure present in a Pickering emulsion. CRL accumulated in multilayers at the droplet interface. For positively charged particles, attractive interactions with CRL led to a 30% higher enzyme packing density and thicker interfacial layers (0.75 μm) compared to negatively charged particles (0.51 μm) at pH 8.3. The looser structure observed with negatively charged particles may account for the formation of smaller droplets. These findings further supplement the results obtained from the adsorption and rheological measurements at additional energy input. Overall, this work advances the understanding of interfacial layers formed by lipases in the presence of charged nanoparticles, highlighting the role of electrostatic interactions. This insight is crucial for optimizing the catalytic efficiency of lipases in Pickering emulsions and could potentially be extended to other classes of enzymes.

## Supplementary Material



## Notes

Details on particle characterization; supporting Figure for surface pressure of different CRL concentrations; interfacial tension of the octanol/water-interface with varying pH; comparison of the interfacial tension of 0.5 wt % particles with different purity grades of octanol; interfacial tension of unmodified silica particles at 0.5 wt % in the aqueous phase for different pH; detailed summary of parameters obtained from interfacial amplitude sweep; image of observed particle sedimentation after measurement; image of particle dispersion with different amount of water added; graphs showing sedimentation behavior of the particles over time; CLSM images of Pickering emulsions without enzyme; measurement of the layer thickness of spontaneously adsorbed CRL at the interface; scatter-plots of all droplets analyzed; comparison of the interfacial tension of labeled and unlabeled CRL (PDF)
