# Supplementary material for: Effect of pH and Particle Charge on the Interfacial Properties of Biocatalytic Pickering Emulsions—Where Are the Enzymes Located?
Source: Langmuir. 2025 Sep 8;41(37):25264–76. doi: 10.1021/acs.langmuir.5c02459 (PMC12461932; doi:10.1021/acs.langmuir.5c02459)
Supplement: Supplementary file 1 [file la5c02459_si_001.pdf]

Supporting Information

Effect of pH and particle charge on the interfacial properties of biocatalytic Pickering emulsions - Where are the enzymes located?

Maximilian Seiler[1,2]*,Maria Loidolt-Krüger[3], Regine von Klitzing[4], Anja Drews[2]

[1]Department of Chemical and Process Engineering, Technical University Berlin, Ackerstraße 76, 13355 Berlin, Germany

[2]Process Engineering at Life Science Engineering, HTW Berlin, Wilhelminenhofstraße 75 A, 12459 Berlin, Germany

[3]Picoquant, Rudower Chaussee 29 (IGZ), 12489 Berlin, Germany

[4]Department of Physics, Soft Matter at Interfaces, Technical University Darmstadt, 64289 Darmstadt, Germany

*Email: maximilian.seiler@htw-berlin.de

Number of pages: 11
Number of figures: 10
Number of tables: 2

Table of contents

Table S1: Details on particle characterization **S2**

Figure S1: Surface pressure of different CRL concentrations **S2**

Figure S2: Interfacial tension of the octanol/water-interface with varying pH **S3**

Figure S3: Comparison of the interfacial tension of particles with different purity grades of octanol.......... **S3**

Figure S4: Interfacial tension of unmodified silica particles in the aqueous phase for different pH.......... **S4**

Table S2: Summary of parameters obtained from interfacial amplitude sweep **S5**

Figure S5: Image of observed particle sedimentation after measurement.......... **S5**

Figure S6: Image of particle dispersion with different amount of water added **S6**

Figure S7: Sedimentation behavior of the particles over time.......... **S7**

Figure S8: CLSM images of Pickering emulsions without enzyme **S8**

Figure S9: Measurement of the layer thickness of spontaneously adsorbed CRL at the interface **S9**

Figure S10: Scatter-plots of all droplets analyzed.......... **S10**

Figure S11: Comparison of the interfacial tension of labeled and unlabeled CRL.. **S11**

Interfacial tension

Table S1: Particle parameters as measured in Stock et al.

	C18n+	C18n-
Sauter mean diameter in nm	27.6 4 ± 3	27.6 ± 3
particle surface area in $m^2\ g^{-1}$	101 ± 10.7	101 ± 10.7
contact angle in $^\circ$	106 ± 8	107.1 ± 4.4
ζ-potential in mV	+53	-50
particle density in $g\ cm^{-3}$	2.15± 0.02	2.15± 0.02

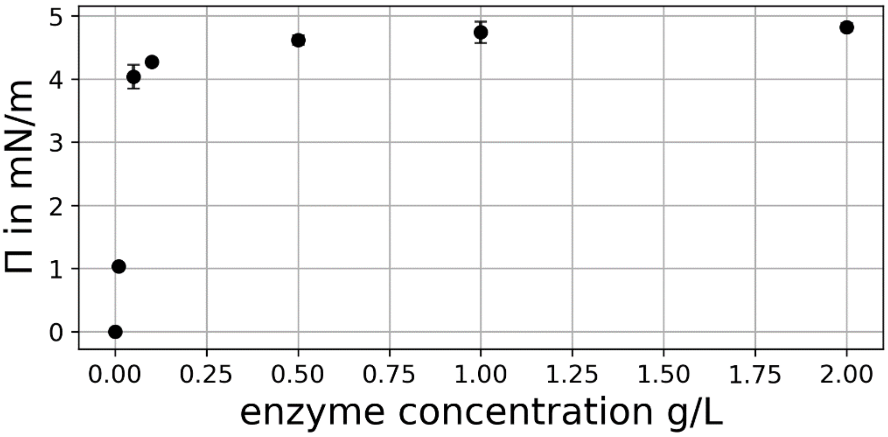

Figure S1: Surface pressure of CRL at pH 7 (100 mM) for varying enzyme concentration.

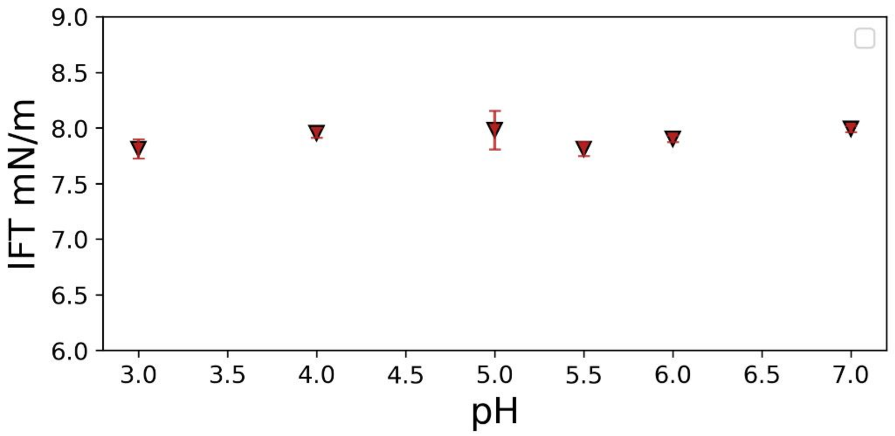

Figure S2: Interfacial tension of the buffer/octanol interface of different pH. Phosphate buffers for pH 6 and 7 (100 mM) and citrate buffers (100 mM) between pH 3 and 5.5 were used.

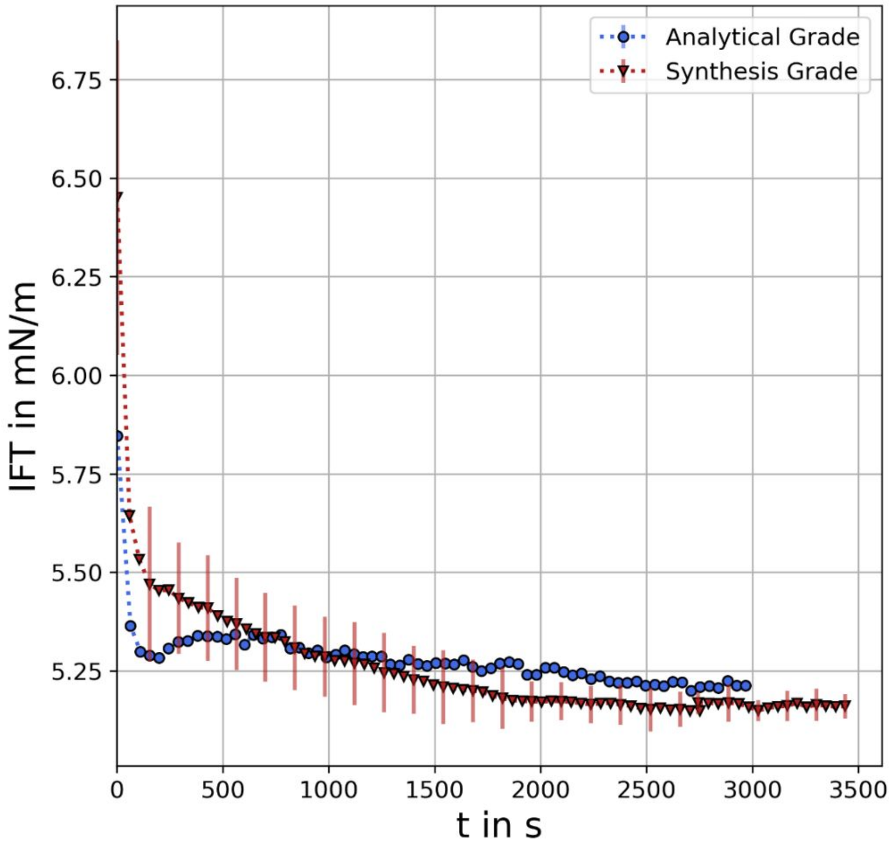

Figure S3: Comparison of the influence of different 1-octanol purities on the interfacial tension of the buffer/octanol at pH 7 with 0.5 wt% positively charged particles.

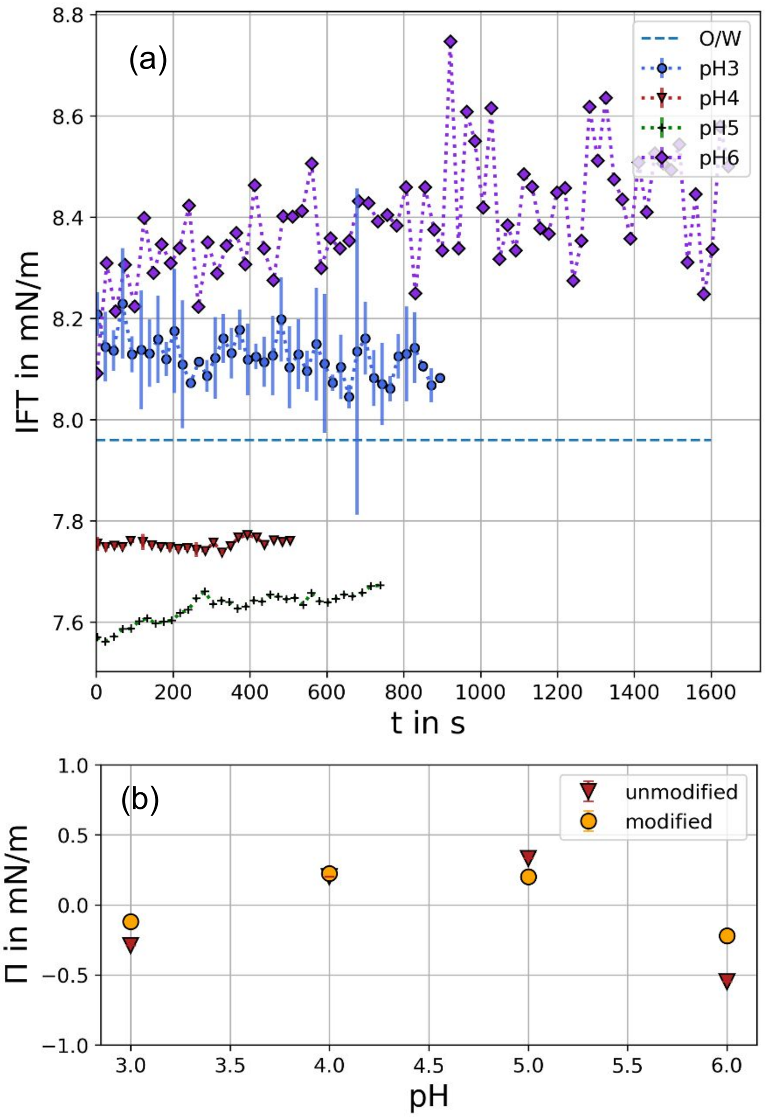

Figure S4: (a) Interfacial tension of 0.5 wt% Ludox-particles in phosphate buffer (100 mM) at the octanol-water interface for different pH values. (b) Respective mean surface pressure.

Interfacial shear rheology

Table S2: Summary of parameters gained from the amplitude sweep.

	pH	$G'_i mPa$	STD	γ_f	STD	γ_y
CRL	3	10.82	0.41	13.9	0.33	1.86
	7	3.396	0.36	7.07	0.2658	2.17
NP +	3	0.69	0.19	6.6	0.146	0.069
	7	0.77	0.33	6.97	0.258	0.542
NP -	3	0	0	0	0	0
	7	0	0	0	0	0
CRL + NP +	3	10.992	0.934	10.78	0.472	1.01
	7	15.04	2.28	18.1	0.75	1.37
CRL + NP -	3	12.56	1.62	18.39	1.18	2.17
	7	5.38	1.4	6.45	0.576	1.37

Particle Sedimentation behavior

Slight clouding at the interface after roughly 48 hours were observed for positively charged particles and after 24 hours for negatively charged particles during interfacial rheology measurements (see Figure S5).

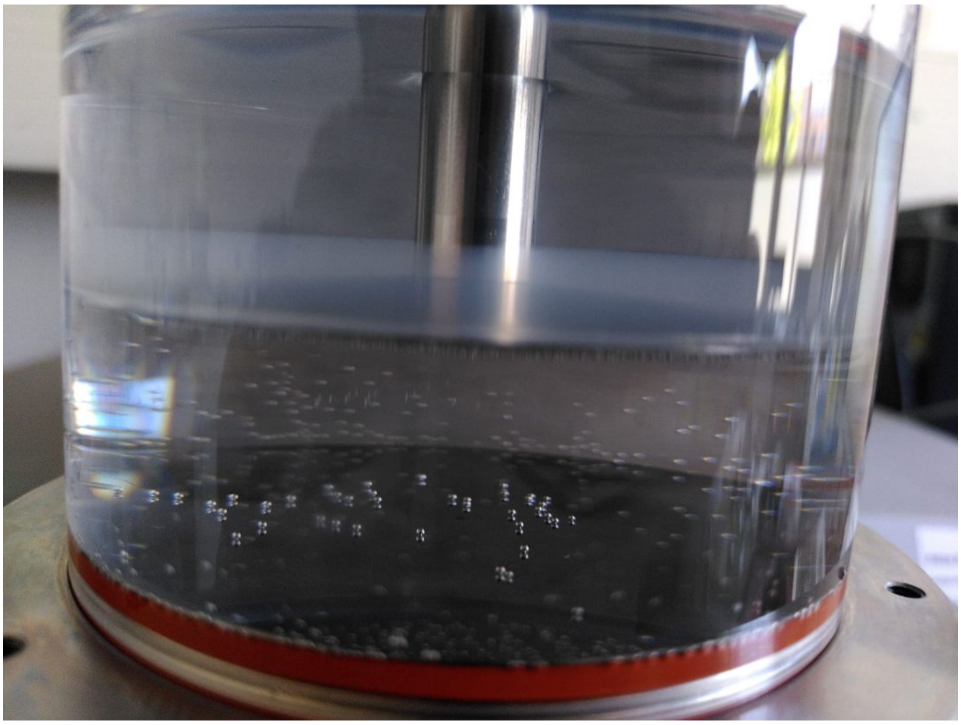

Figure S5: Sedimentation of negatively charged silica particles could be observed after approximately 24 h.

To further quantify the rate of aggregation and sedimentation behavior, the particle concentration in octanol was measured over time. This was done by measuring the absorption of the nanoparticles dispersed in octanol at 215 nm with an UV-VIS spectrometer UV-1900 (Shimadzu, Kyoto, Japan).

The dissolved water content was determined by Karl Fischer titration using an Eco Coulometer (Metrohm, Filderstadt, Germany).

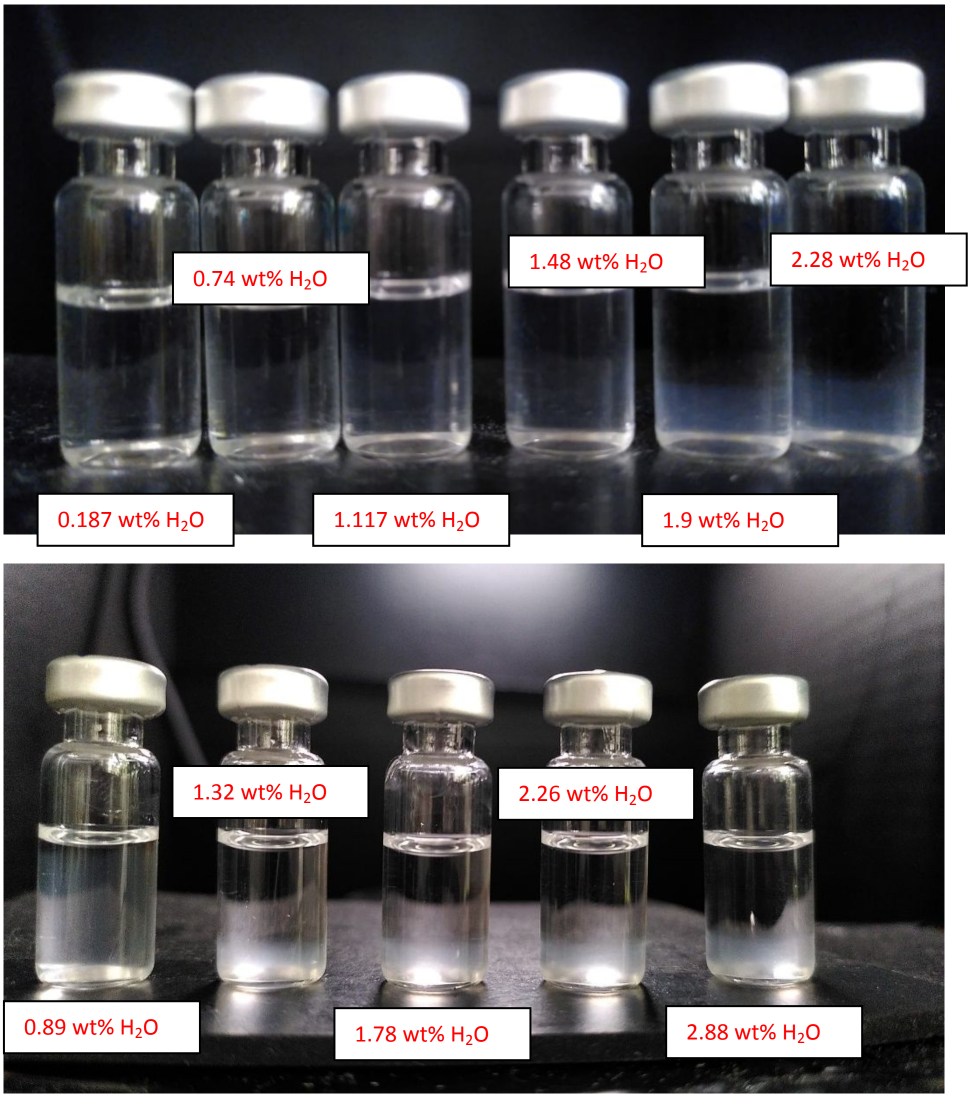

Figure S6: 0.5 wt% positively charged nanoparticles (top) and negatively charged particles (bottom) dispersed in octanol three days after preparation. Different amounts of phosphate buffer (pH7, 100 mM) were added to observe the effect of dissolved water on sedimentation. Clouding at the bottom can be seen at higher water content, above 1.9 wt% for positively charged particles and at 0.89 wt% for negatively charged particles.

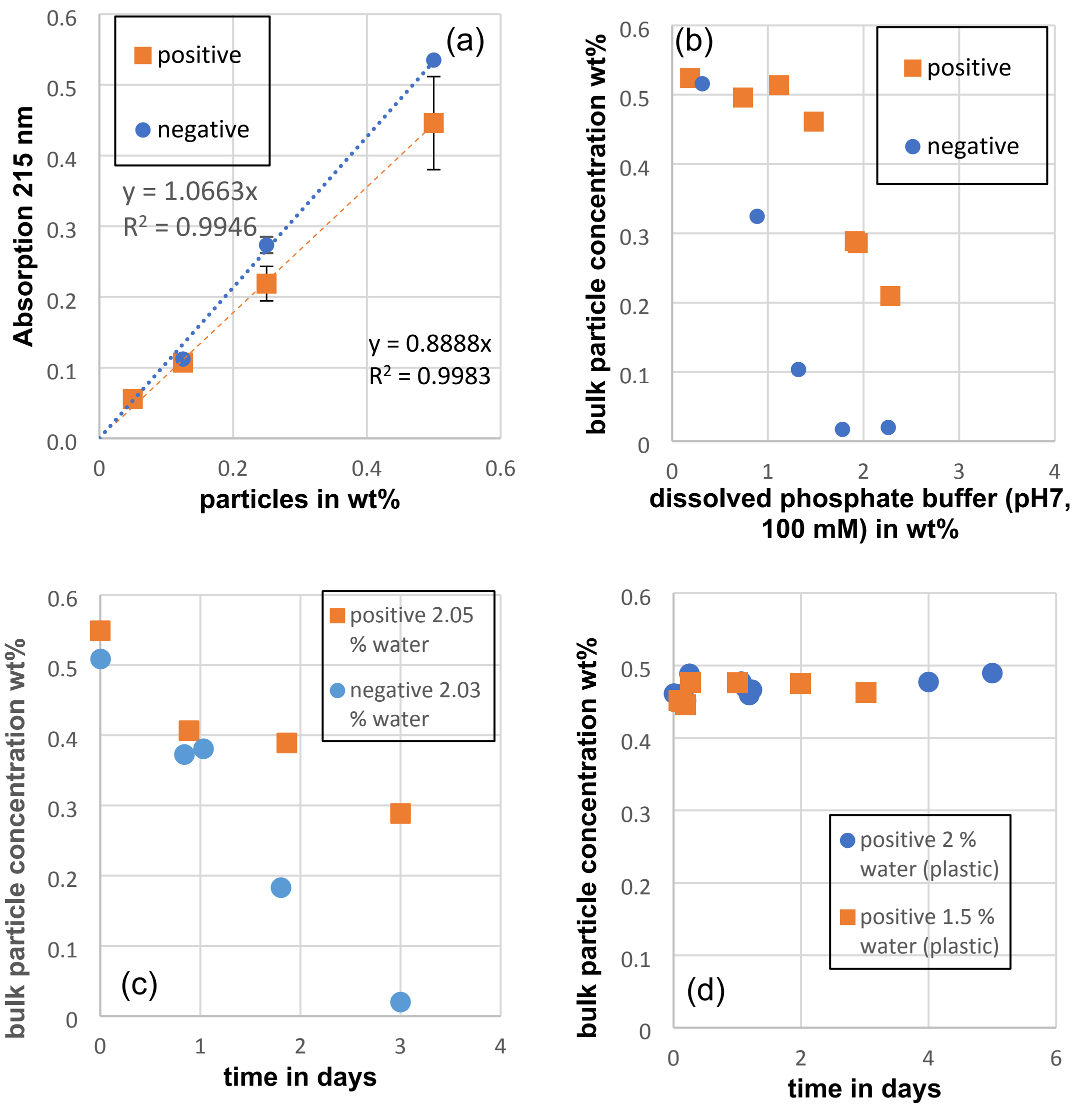

Figure S7: (a) Calibration curve for the absorption of positively and negatively charged particles at 215 nm. Particles were dispersed in dry octanol (0.03-0.06 wt% water). (b) Particle amount as a function of added phosphate buffer (pH7, 100 mM). For measuring the adsorption, the upper 0.5 mL were carefully pipetted into a cuvette. Measurements were taken three days after preparation. (c) Time-dependent sedimentation measurements at a water content of 2 wt%. The particle dispersions were prepared and kept in glass tubes. (d) Analogous time-dependent sedimentation measurements of positively charged particles using dispersions in plastic tubes. Sedimentation did not occur, when particles were stored in a plastic tube.

Preparation of fluorescent Pickering emulsion

With a molar mass of 58.954 *kg/mol* (LIP1_DIURU), 1 *mL* of enzyme solution with a concentration of 0.5 *g/L* results in 8.75 *nmol*. The dye has a molar mass of 288 *g/mol*. With a concentration 1 *g/L*, 2.53 *µL* would provide an equimolar amount.

A small fluorescence signal is emitted by the nanoparticles as seen Figure S8 (a) and (b). This fluorescence locates the particles mostly at the interface of the droplets. The intensity values reach only about 5 % of the signal emitted by the labeled lipase.

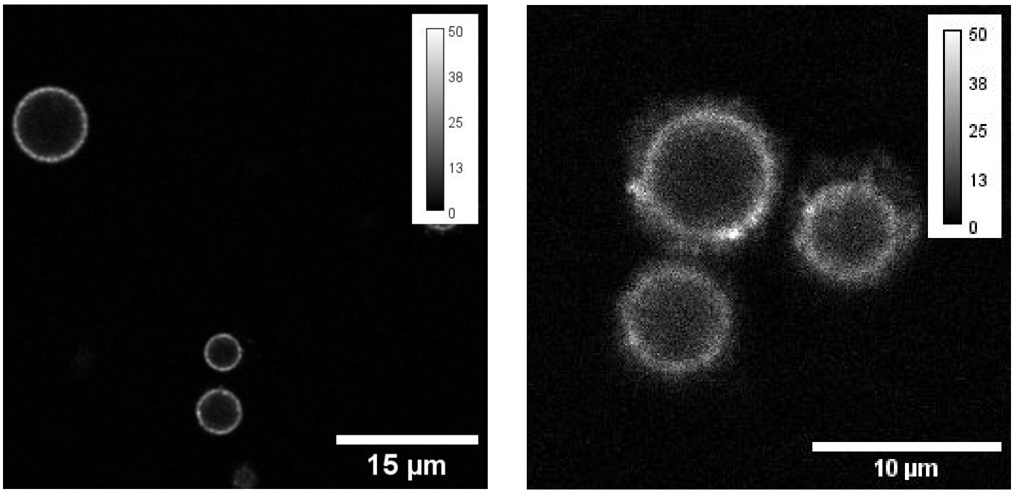

(a) unlabeled, negative (b) unlabeled, positive

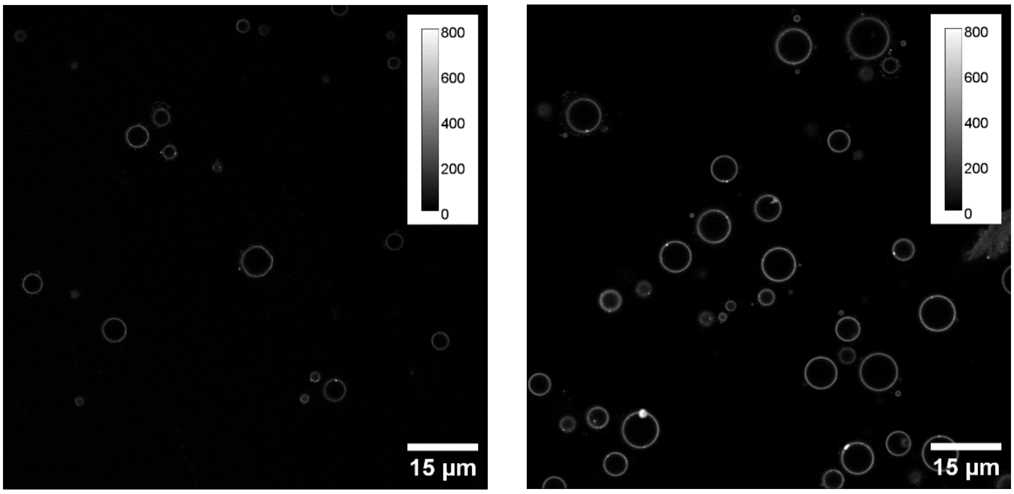

(c) labeled, negative (d) labeled, positive

Figure S8: Comparison of the fluorescent signal of droplets without labeled lipase with negatively charged (a) and positively charged (b) particles. As comparison Pickering emulsions prepared with labeled lipase and negatively charged (c) or positively charged particles (d). The calibration bar shows the brightness of each pixel.

Emulsions prepared without particles are not kinetically stable, exhibiting rapid phase separation and irregular droplet formation. To measure the interfacial layer thickness in the absence of particles, a droplet of labeled enzyme solution (~5 µL) was placed on a microscope slide and covered with octanol. To assess potential time dependence, images were captured at various time points and analyzed as described in the Methods section.

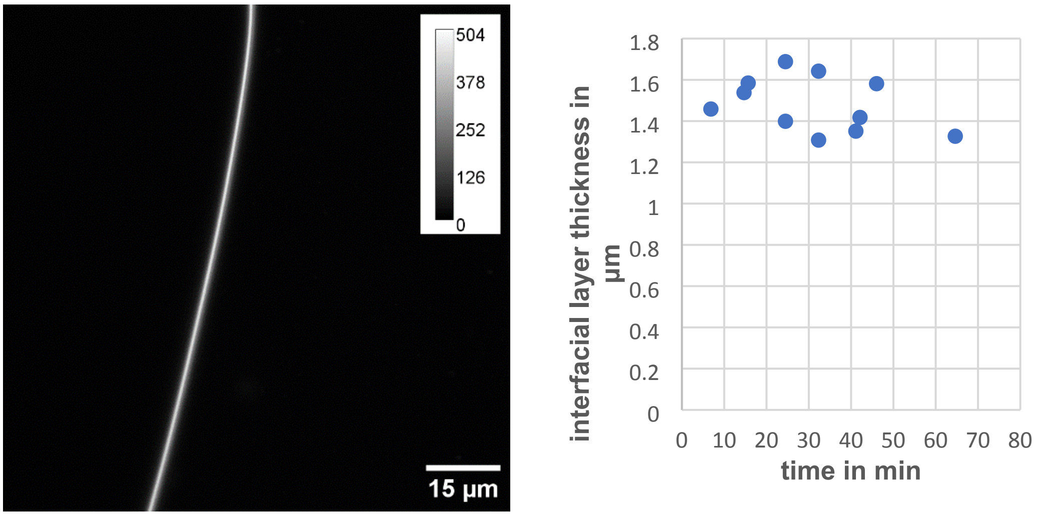

(a) (b)

Figure S9: (a) Labeled CRL at the octanol-water interface without the addition of particles. (b) Interfacial layer thickness at different locations imaged at different time points.

An average interfacial layer thickness of 1.48 µm ($\pm$ 0.13 µm) and a corresponding average peak concentration of 1.27 g L^{-1} ($\pm$ 0.425 g L^{-1}) were observed for CRL at the octanol-water interface.

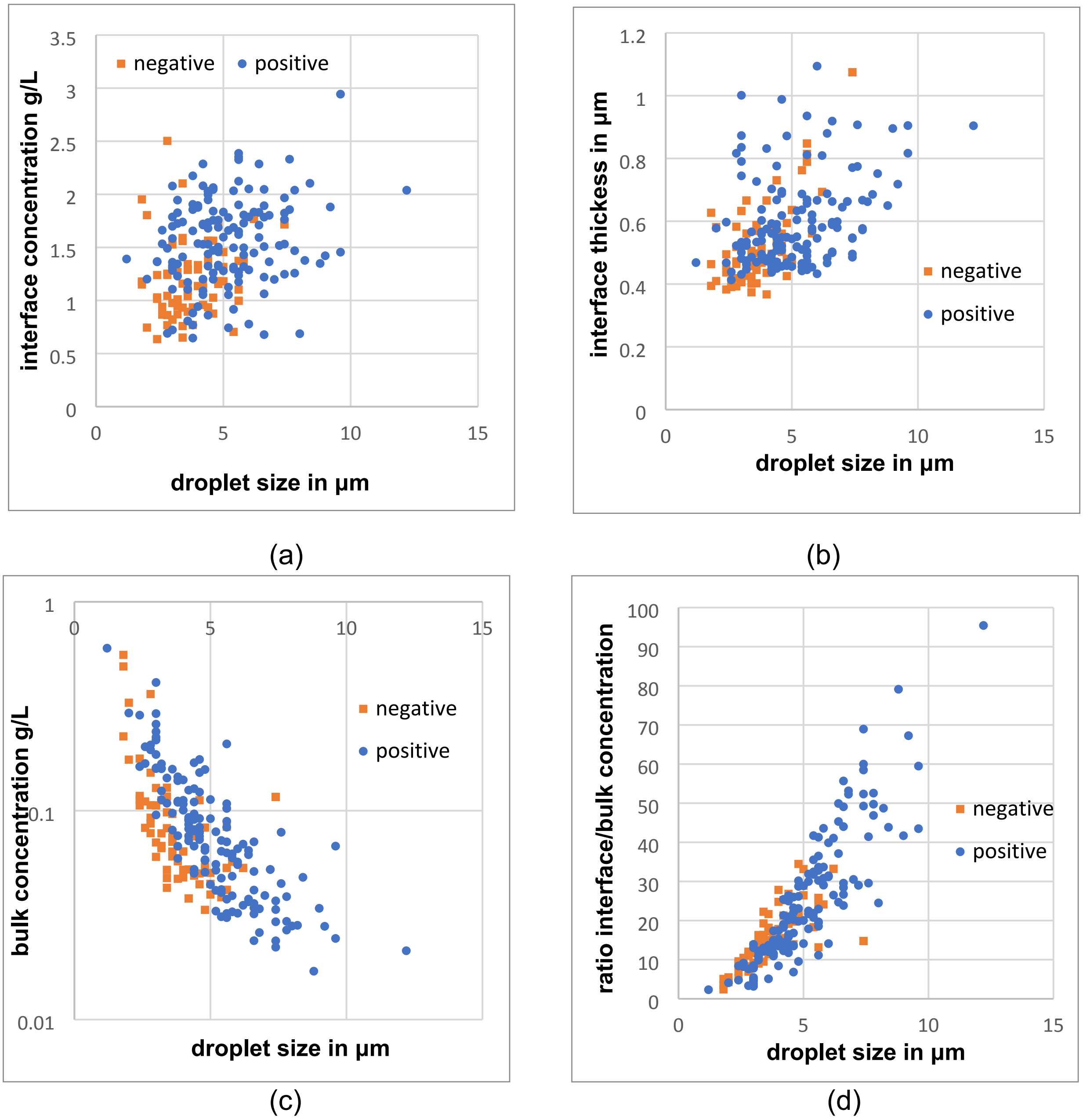

Figure S10: (a) Interface concentration, (b) interface thickness, (c) bulk concentration and (d) the ratio between interface and bulk concentrations of all analyzed droplets in dependence of droplet size.

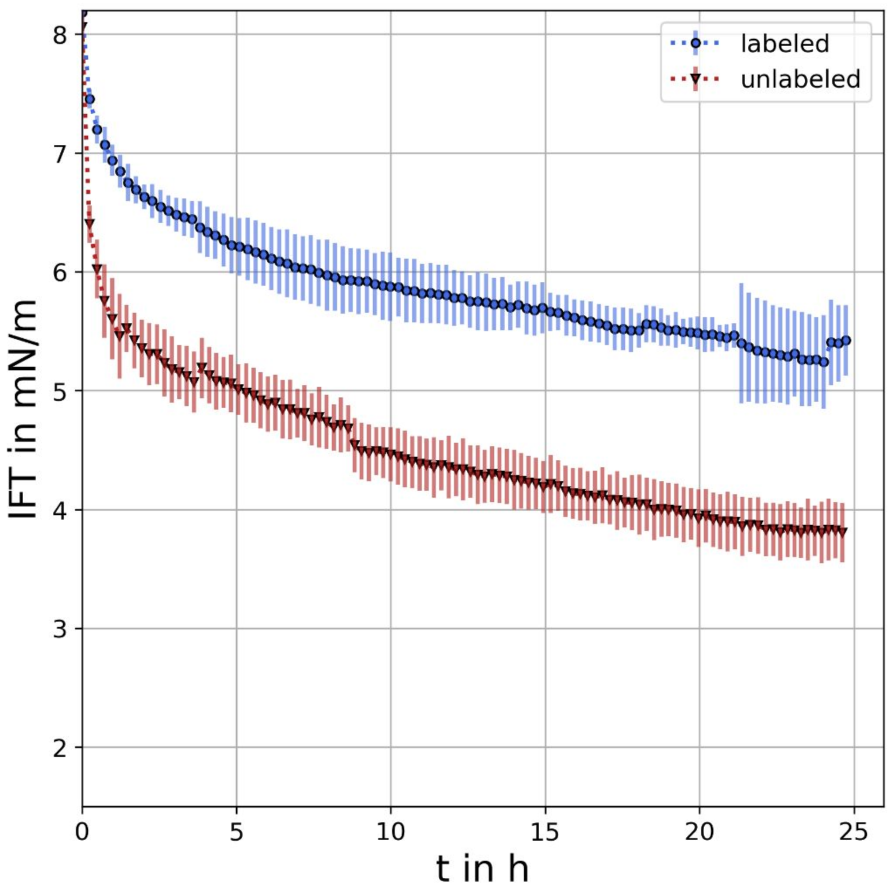

Figure S11: Adsorption behavior of CRL after labeling. Measurements were performed with a w/o pendant drop (9 μL). Enzyme solution were prepared with 0.5 g/L labeled/unlabeled lipase in phosphate buffer (pH8.3, 100 mM). Octanol was saturated with the aqueous phase prior to measurement. The dye itself showed no surface activity. Experiments were repeated at least three times.
